# Putative Contribution of 8-Aminoquinolines to Preventing Recrudescence of Malaria

**DOI:** 10.3390/tropicalmed8050278

**Published:** 2023-05-15

**Authors:** Miles B. Markus

**Affiliations:** 1 Wits Research Institute for Malaria, School of Pathology, Faculty of Health Sciences, University of Witwatersrand, Johannesburg 2193, South Africa; miles.markus@wits.ac.za; 2 School of Animal, Plant and Environmental Sciences, Faculty of Science, University of Witwatersrand, Johannesburg 2001, South Africa

**Keywords:** bone marrow, hydrogen peroxide, hypnozoite, malaria, *Plasmodium vivax*, primaquine, recrudescence, relapse, spleen, tafenoquine

## Abstract

Enhanced therapeutic efficacy achieved in treating *Plasmodium vivax* malaria with an 8-aminoquinoline (8-AQ) drug such as primaquine (PQ) together with a partner drug such as chloroquine (CQ) is usually explained as CQ inhibiting asexual parasites in the bloodstream and PQ acting against liver stages. However, PQ’s contribution, if any, to inactivating non-circulating, extra-hepatic asexual forms, which make up the bulk of the parasite biomass in chronic *P. vivax* infections, remains unclear. In this opinion article, I suggest that, considering its newly described mode of action, PQ might be doing something of which we are currently unaware.

## 1. Introduction

The 8-aminoquinolines (8-AQs) primaquine (PQ) [1] and tafenoquine (TQ) [2] are likely to become very important for controlling *Plasmodium vivax* malaria [3,4,5]. This is the type of human malaria [6] that is the most widespread globally [7], and approximately 2.5 billion people are potentially at risk of contracting the disease. Unsurprisingly, a higher total dose of PQ [8,9,10,11,12], or a larger single dose of TQ [13,14,15], is more therapeutic than a lower dose, but how these drugs work in preventing recurrent *P. vivax* malaria is far from clear. The question of what effective 8-AQ dosages are safe to use is still being researched [14,16,17,18,19], as is how to distinguish between *P. vivax* malarial reinfections, recrudescences and relapses [20].

At present, it is widely believed that, in many geographical areas, the majority of non-reinfection *P. vivax* malarial recurrences are relapses. The origin of relapse in malaria is by definition hepatic hypnozoite activation [21]. What precipitates this activation remains unknown, although there are various theories [22]. If hypnozoite-like plasmodial stages also persist outside the liver, which is a possibility parasitologically [23,24], this has yet to be discovered. Figures of up to well over 80% for the proportion of recurrences that are thought to be relapses can be found in the literature (see [25]). These estimates are derived largely by extrapolating from the results of treatment that included PQ, which is routinely co-administered with a recognised blood schizontocidal agent (following Peters [26], I use the suffix “-ocidal” here, instead of “-icidal”). This drug-related determination of approximate relapse frequency is not necessarily correct. My question in this paper, reflected by the title, is: does the use of 8-AQs result in significant suppression of blood-stage parasites in addition to the inactivation of liver stages? It is a drug-associated question that is inextricably linked to theories about what *P. vivax* stages are the origin of recurrences (Table 1), because some conclusions about drug efficacy have been based on proven or unproven aspects of *P. vivax* biology.

Despite the general acceptance of the assertion that most non-reinfection episodes of recurrent *P. vivax* malaria are relapses, it is nevertheless unclear to me why hypnozoites should be the origin, in many human communities, of such a large number of non-reinfection recurrences. It does seem feasible, however [25,27,28,29]. Alternatively, non-circulating blood-stage merozoites (i.e., merozoites not detectable in peripheral blood) might be the source of more recrudescences (as opposed to relapses) than is readily apparent [30,31]. The latter possibility is not a new suggestion, but a long-standing idea (Table 1) supported by the fact that intra-erythrocytic stages have, in the meantime, been shown to be hidden outside the peripheral circulation in vast numbers (compared to what must be relatively few hypnozoites) in individuals chronically infected by *P. vivax* [32,33,34,35,36,37]. Parasitologically, therefore, it is not so much a question of why such merozoites would often be the source of recrudescent *P. vivax* malaria, but rather why they would not be. I see these concealed asexual parasites as a threat to achieving the goal of eliminating malaria [38], as is now agreed on by others [39,40,41,42,43].

**Table 1:** Relapse in malaria: main related events and hypotheses ^1^.

Year	Details	Reference(s)
Pre-1948	The most prevalent theory before 1948 ascribed the origin of relapses to parasites in the reticulo-endothelial system	[44]
1948	Discovery of hepatic schizogony in the life cycle of primate *Plasmodium*. This led to malarial relapse being explained as the consequence of ongoing cycles of schizogony taking place in the liver (assumed to be the source of parasites for renewed erythrocytic schizogony)	[45]
1976	Discovery of the apicomplexan hypnozoite (non-malarial) by ultrastructural recognition of its sporozoite-like nature	[46]
1976	Occurrence of hypnozoites in the life cycle of *Plasmodium* predicted on the basis of non-plasmodial research results (by extrapolation)	[47]
1978	Coining of the term “hypnozoite” and its adoption for *Plasmodium* (in advance of and in anticipation of the future discovery of malarial hypnozoites)	[48,49]
1980	Discovery of the malarial hypnozoite, resulting in the hypnozoite hypothesis of relapse and latency in malaria becoming established	[50]
2011	First proposal in the post-hypnozoite-discovery era that there might be one or more hypnozoite-independent, non-bloodstream sources of homologous (specifically) *Plasmodium vivax* parasites in recurrences. Such recurrences would be recrudescences, not relapses. The suggestion is that *P. vivax* malarial recurrences are being over-attributed to hypnozoite activation	[51,52,53]

^1^ Reproduced in modified form, with the publisher’s permission, from Markus, M.B. Biological concepts in recurrent *Plasmodium vivax* malaria. *Parasitology* **2018**, *145*, 1765–1771 [30]. © Cambridge University Press, 2018.

In this opinion piece, I refer to known intra-erythrocytic plasmodial parasite inactivation by the two currently used 8-AQ drugs, especially PQ. I also refer, more specifically, to the consequence of exposure of these stages to hydrogen peroxide (H_2_O_2_), which can be deleterious for cells at above-physiological levels [54]. This is important because, normal H_2_O_2_ involvement in malaria aside [55,56], one of perhaps more places where H_2_O_2_ apparently accumulates as a result of PQ administration is in the bone marrow [57], a habitat where *P. vivax* is not readily detectable and in which it thrives [33,34,37].

## 2. Inactivation of Intra-Erythrocytic Stages of *Plasmodium* by Primaquine

The fact that PQ, whether administered alone or with a partner drug, can act against extra-hepatic, asexual plasmodial parasites has received little consideration lately. The elimination of gametocytes in vitro [58] or in vivo by PQ and the prophylactic usage thereof aside, the drug is, otherwise, usually only thought of as a hypnozoitocidal agent and one which also inactivates schizonts in the liver [59]. This hitherto presumed contribution to the radical cure of *P. vivax* malaria has now actually been demonstrated microscopically in vivo, albeit in humanized mice [60]. These findings follow the confirmation by Voorberg-van der Wel et al. that hypnozoites in liver cells do in fact activate [61,62], as had been assumed for decades (Table 1). Only rarely in this millennium have malariologists, quite rightly, expressed caution about dogmatically making this assumption [63,64] without such hard scientific evidence.

The rest of this section is a summary of mostly early research examples of the effect of PQ on intra-erythrocytic plasmodial stages. Section 5 provides additional information, based mainly on later work. 

Arnold et al. [65] researched PQ monotherapy with respect to asexual blood stages in six male, non-immune human volunteers following infection with the Pf6 Panamanian strain of *P. falciparum*. For unknown reasons, the response to PQ varied clinically and as regards asexual parasitaemia. Although one individual responded well to 30 mg of PQ base daily for 14 days, a recrudescence occurred after this period. The authors concluded that, overall, PQ had a poor antimalarial effect in their study subjects and that treatment of *P. falciparum* malaria with PQ alone would be inappropriate.

An in vivo study which often seems to be overlooked is one carried out in Thailand on the clearance of bloodstream parasitaemia. It was found that *P. vivax* malaria responded well initially when PQ was administered alone to patients [66]. This suggests to me that PQ might have some effect on non-circulating, intra-erythrocytic parasites such as in the spleen or bone marrow.

Basco et al. [67] assessed the in vitro blood schizontocidal effect of PQ on fresh *P. falciparum* clinical isolates obtained from patients in Cameroon. PQ was less active against asexual stages than some standard blood schizontocides, but more effective than antibiotics that have been used for treating malaria, such as clindamycin and doxycycline.

Various authors have found PQ to be more potent in vitro against CQ-resistant *P. falciparum* than against CQ-sensitive parasites, e.g., [68].

## 3. Blood Schizontocidal Action of Tafenoquine

The effective action of TQ against blood-stage plasmodial parasites has been described in a number of publications, including those mentioned here and in Section 5, below.

For example, isolates of *P. falciparum* from widely separated localities in Africa were found to be highly susceptible to TQ exposure in vitro, and baseline susceptibility has been defined [69]. Although other publications also report good efficacy in vitro, low activity of TQ was noted in a study using the 3D7 strain of *P. falciparum* [70]. There is evidence that malaria parasites are more susceptible to TQ in vivo than in vitro [71].

In vivo, TQ is by far superior to PQ as regards its inhibitory effect on asexual parasitaemia in various kinds of rodent malaria [72], while findings in *P. vivax*-infected *Aotus* monkeys revealed that TQ might be useful for treating chloroquine-resistant *P. vivax* malaria [73]. These diverse experimental situations aside, Barber et al. [15] have demonstrated that TQ has potent antimalarial activity in human volunteers infected with the 3D7 strain of *P. falciparum*, but efficacy is dose-dependent.

In the light of these findings and others, e.g., [74], it seems possible that TQ inactivates not only asexual parasites in the peripheral circulation, but also non-circulating, intra-erythrocytic parasites that are hidden elsewhere in the body.

## 4. Effect of Hydrogen Peroxide on *Plasmodium*

We need to ask whether the inclusion of PQ in the drug therapy of patients with *P. vivax* malaria might facilitate H_2_O_2_-associated inactivation of non-circulating asexual stages, thereby preventing recrudescences [75]. Research involving H_2_O_2_ indicates that this possibility should be investigated. However, there is not much information on the effect of H_2_O_2_ on *Plasmodium*, and more assays need to be carried out.

The intravenous injection of H_2_O_2_ suppressed parasitaemia in *P. vinckei*-infected mice, with degenerated intra-erythrocytic parasites being recovered from the bloodstream [76]. Other researchers [77] reported that both *P. berghei* and *P. yoelii* blood stages were killed in vitro by even low concentrations of H_2_O_2_. They also found that the intravenous injection of a tolerable dose of H_2_O_2_ into mice of two strains gave identical results, namely a marked reduction in *P. chabaudi* and *P. yoelii* parasitaemia. However, *P. berghei* was less susceptible in vivo.

As regards oxidative stress in relation to the possible mechanism of action of some compounds used for treating malaria, van Schalkwyk et al. [78] showed for *P. falciparum* in vitro (3D7, D10 and Dd2 strains) that exposure to H_2_O_2_ caused the parasite’s adenosine triphosphate level to drop. This was accompanied by a marked disturbance of intracellular pH regulation, namely acidification of the parasite’s cytosol and alkalinisation of the digestive vacuole. In the experimental system of Wezena et al. [79], using *P. falciparum* strains 3D7 and Dd2, parasite killing required high concentrations of H_2_O_2_.

Utaida et al. [80] determined that H_2_O_2_ in combination with some antibiotics markedly inhibited *P. falciparum* parasites of the K1 strain. This led the authors to comment that “by a judicious choice of drug combinations it should be possible to obtain beneficial anti-plasmodial drug partners of otherwise non-efficacious antimalarials”. The co-administration of PQ and CQ comes to mind as a possible example hereof in some instances.

In the bone marrow, would an inhibitory effect of H_2_O_2_ be via damage to the intra-erythrocytic parasites, or their host cells, or both [81]? A single-cell approach demonstrated morphologically detectable damage to erythrocytes caused by H_2_O_2_ and PQ [82]. Moreover, it was shown that *P. falciparum* (3D7 strain) was unable to penetrate into cells that had such cytoskeletal damage and increased membrane stiffness [82].

## 5. Drug Combinations and Modifications

Many researchers have noted enhanced anti-plasmodial activity in vivo [83,84] or in vitro when 8-AQs were combined with other drugs rather than used alone. This includes the first detailed microscopic examination of the in vivo effect on hypnozoites and hepatic schizonts of PQ combined with chloroquine (CQ), as well as of a three-drug combination [60]. The illustrative examples given here, for both dual drug therapy and hybrid compounds, include instances of this efficacy enhancement (in addition to the related information provided above). Together, the details below cover drug effects on *P. falciparum*, *P. vivax* and rodent malarial parasites.

PQ has for a long time normally been regarded as a weak blood schizontocide [85]. Nonetheless, established laboratory strains of *P. falciparum* or fresh clinical isolates have repeatedly been used to see what happens when blood stages are subjected to dual PQ or TQ and partner schizontocide exposure. Depending on the drug combination and the parasite strain, interactions in vitro have frequently been synergistic and sometimes additive or antagonistic [86,87,88,89,90,91,92]. Amongst TQ-partner drug combinations, TQ-methylene blue was especially synergistic against *P. falciparum* in vitro [90].

Baird et al. [93] suggested that some of their human treatment findings could perhaps be explained as the combination of PQ and CQ being more effective than CQ alone against intra-erythrocytic *P. vivax* in patients harbouring CQ-resistant parasites. The possibility of increased blood-stage asexual parasite inhibition has also been taken into account in other *P. vivax* studies where PQ was co-administered, e.g., [94]. The mechanism by which asexual blood stages are seemingly inactivated via this enhancement is a matter for speculation.

Chemical research has been carried out with a view to improving the blood-stage antimalarial efficacy of PQ [95], with many PQ derivatives having been prepared and tested for their overall antiplasmodial activity [96,97]. 8-AQ hybrid compounds might become very relevant for malaria eradication if they act against non-circulating, intra-erythrocytic asexual forms, which are probably a hindrance to achieving the aim of eradication [38]. The covalent linkage of PQ and artemisinin did indeed result in marked efficacy of a compound against plasmodial blood stages [98], as did some non-artemisinin-associated 8-AQ hybrids [99]. A PQ-pyrimidine hybrid has been described as having good blood-stage antiplasmodial activity [100]. The strategy of using metallic hybrid-based drugs was designed [101] in line with the standard treatment of *P. vivax* malaria with a combination of PQ and CQ, and both metallic and non-metallic PQ-CQ hybrid compounds have proved to have significant inhibitory effects on blood-stage *Plasmodium* parasites [102,103]. Some such 8-AQ hybrid antimalarials, and others [104], inhibit intra-erythrocytic, multidrug-resistant *P. falciparum*.

In practice, the most common combination treatment currently given to patients with *P. vivax* malaria is PQ + CQ. However, when CQ-resistance has been suspected, an alternative companion drug to PQ has been tried, e.g., [105].

## 6. Conclusions

The effect of 8-AQ antimalarials on non-circulating asexual stages of *P. vivax*, such as in the bone marrow [106,107], needs to be investigated, including whether 8-AQs or their partner drugs can induce temporary quiescence of intra-erythrocytic *P. vivax* parasites, a subject that has been raised elsewhere [30,63]. It is conceivable that the apparently increased in vivo schizontocidal activity in peripheral blood that results when therapy includes both PQ and CQ [84] also reflects increased inactivation of non-circulating asexual parasites, thereby preventing recrudescences. Evidence one way or the other should soon be forthcoming from drug-related experimentation using humanized mice [108].

## Data Availability

Not applicable.
